# New insight of complement system in the process of vascular calcification

**DOI:** 10.1111/jcmm.17732

**Published:** 2023-04-01

**Authors:** Aiting Liu, Pei Luo, Hui Huang

**Affiliations:** ^1^ Department of Cardiology, The Eighth Affiliated Hospital, Joint Laboratory of Guangdong‐Hong Kong‐Macao Universities for Nutritional Metabolism and Precise Prevention and Control of Major Chronic Diseases Sun Yat‐sen University Shenzhen China; ^2^ State Key Laboratory for Quality Research in Chinese Medicines Macau University of Science and Technology Macau China

**Keywords:** cardiovascular diseases, complement system, vascular calcification

## Abstract

The complement system defences against pathogenic microbes and modulates immune homeostasis by interacting with the innate and adaptive immune systems. Dysregulation, impairment or inadvertent activation of complement system contributes to the pathogenesis of some autoimmune diseases and cardiovascular diseases (CVD). Vascular calcification is the pivotal pathological basis of CVD, and contributes to the high morbidity and mortality of CVD. Increasing evidences indicate that the complement system plays a key role in chronic kidney diseases, atherosclerosis, diabetes mellitus and aging‐related diseases, which are closely related with vascular calcification. However, the effect of complement system on vascular calcification is still unclear. In this review, we summarize current evidences about the activation of complement system in vascular calcification. We also describe the complex network of complement system and vascular smooth muscle cells osteogenic transdifferentiation, systemic inflammation, endoplasmic reticulum stress, extracellular matrix remodelling, oxidative stress, apoptosis in vascular calcification. Hence, providing a better understanding of the potential relationship between complement system and vascular calcification, so as to provide a direction for slowing the progression of this burgeoning health concern.

## 1 INTRODUCTION

Vascular calcification (VC) is the key pathological basis of cardiovascular diseases (CVD), and contributes to the high morbidity and mortality of CVD. Initially, VC was thought to be the result of passive degenerative processes, but cumulative researches have proved that VC is an active, multifactorial and highly regulated process.^1^ Although cumulative published studies have explored various mechanisms of VC, there are still no effective drugs to delay the process of VC.

**FIGURE 1:**
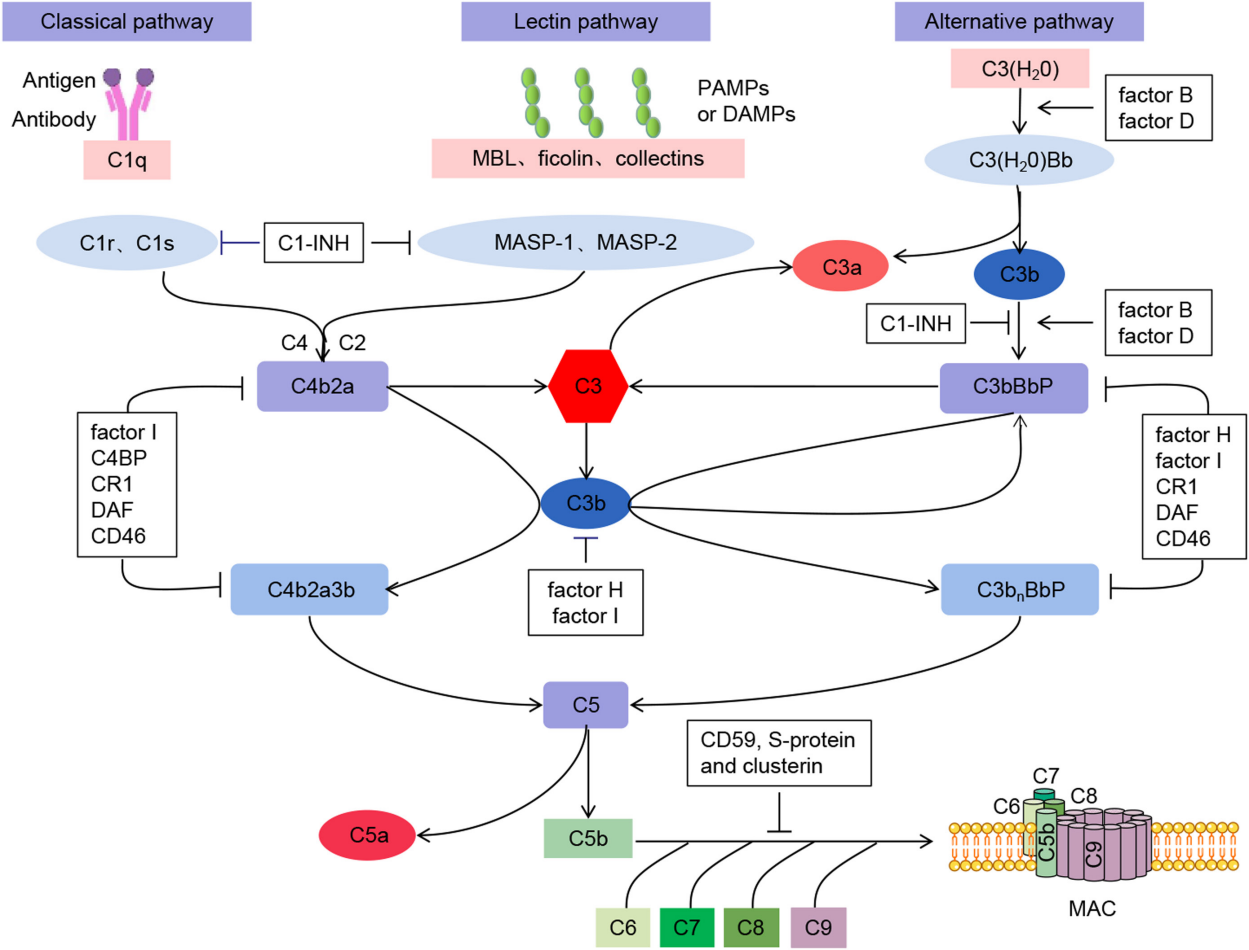
The activation of complement system. The complement system is activated by the classical, lectin and alternative pathways. Complement component C1q activates the classical pathway by binding to antigen–antibody complexes. The lectin pathway is activated by MBL, ficolins and collectins interacting with PAMPs or DAMPs. Activation of the lectin and classical pathways generates C3 convertase (C4b2a). The alternative pathway is activated by low grade spontaneous hydrolysis of systemic native C3 [C3(H_2_O)] or via properdin binding to certain cell surfaces, and then the C3 convertase (C3bBb), stabilized by properdin, is generated. The C3 convertases cleave complement component C3 into the anaphylatoxin C3a and the opsonin C3b. Then, C3b combines with C4b2a to activate complement component C5. Finally, C5b, cleaved from C5, participates in the generation of the MAC which inserts into cell membranes and causes cell lysis. In addition, the process of complement activation is regulated by a variety of complement regulatory factors, including C1‐INH, factor H, DAF, C4BP, CR1, DAF, CD46, factor B, factor D, factor I, CD59, S‐protein and clusterin. DAMPs, damage‐associated molecular pattern molecules; MAC, membrane attack complex; MASP, mannan‐binding lectin serine protease; MBL, mannose‐binding lectin; PAMPs, Pathogen‐associated molecular pattern molecules.

Infiltration of immune cells, macrophages, T cells and neutrophils, have been found in the aortic specimens of VC.^2^ Although the underlying mechanism is not fully understood, increasing evidences indicate that the innate immune system plays an essential role in the development of VC. The complement system is the most important part of the innate immune system which is activated by classical, alternative and lectin pathways. It consists of more than 40 membrane and soluble proteins, including plasma complement components, soluble or membrane‐type complement regulatory proteins and complement receptors (Figure 1). The complement system plays a momentous role in various processes, such as removal of senescent cells, tissue remodelling, cell lysis and inflammation.

Cumulative researches have gradually identified that the complement system is closely linked to a number of diseases, including atherosclerosis, aging‐related diseases, diabetes mellitus (DM) and chronic kidney diseases (CKD).^3^, ^4^, ^5^ Notably, VC is prevalent in these diseases. More importantly, an increasing number of osteoimmunological researches have demonstrated that physiological bone mineralisation, similar to the process of VC, is subject to complement modulation.^6^ Taken together, these findings suggest the complement system may take part in VC. As reported, a cross‐sectional clinical study found that C3a was positively correlated with abdominal aortic calcification in patients on haemodialysis,^7^ and this phenomenon was also observed in aortic and coronary calcification among middle aged women.^8^ In addition, the plasma level of C5 was positively correlated with coronary calcification in the PESA study.^9^ Collectively, the complement system may be activated and contribute to the development of VC. However, the association between complement system and VC is less well understood, and the processes are unclear. Therefore, this review summarizes the activation pathways and potential molecular mechanisms of complement system in VC, hence attracting attention to explore whether complement components could be a promising target for VC, and focusing on regulating the balance of complement system in VC.

## 2 THE ACTIVATION OF COMPLEMENT SYSTEM IN VC

The complement system is a surveillance system and is quickly activated by sensing danger in the body, thus helping to maintain tissue homeostasis and promote tissue regeneration and repair. There are three pathways activating the complement system: classical, lectin and alternative. The classical pathway uses C1 and is major triggered by antigen–antibody immune complexes. The lectin pathway uses mannose‐binding lectin (MBL), collectins and ficolins to identify patterns of carbohydrate ligands. Spontaneous hydrolysis of a thioester bond within C3 is thought to initiate the alternative pathway. However, dysregulation, impairment or inadvertent activation of complement system also induces tissue damage and organ dysfunction in the host.

Increasing evidences found the complement system was activated in VC. Lim et al.^10^ aimed to identify proteins associated with progression of aortic stenosis by lipid mass spectrometry imaging and proteomic analysis, and found that aortic calcified microdomains had higher levels of complement components C3, C4b, C5, C8 beta chain and C9, while complement regulatory protein factor H was higher in non‐calcified microdomains. Published researches also found the level of membrane attack complex (MAC) in coronary calcified areas of kidney diseases patients was higher than control subjects.^11^ As we all known, VC is prevalent in CKD, atherosclerosis, DM and aging‐related diseases, and complement system was activated and stimulated the progress of these diseases. Therefore, we summarized the triggers and activation pathway of complement system in VC (Table 1).

**TABLE 1:** The activation of complement system in VC.

Diseases	Trigger for complement activation	activation pathway	References
Chronic kidney diseases	Circulating IgG auto‐antibodies, abnormal deposition of protein in glomerulus, disordered metabolism of calcium and phosphate	Classical pathway	24
		Alternative pathway	20, 21, 22
		Lectin pathway	25
Atherosclerosis	Oxidized low‐density lipoprotein Lipoprotein, enzymatically modified low‐density lipoproteins and cholesterol crystals	Classical pathway	29, 32
		Alternative pathway	31
		Lectin pathway	30
Diabetes mellitus	Circulating IgG auto‐antibodies, hyperglycemia, glycation end products	Classical pathway	37
		Lectin pathway	25, 38
Aging	Inflammation cytokines, reactive oxygen species, N‐glycome	Classical pathway	42, 43
		Alternative pathway	46
		Lectin pathway	44, 45

### 2.1 The activation of complement system in kidney diseases

Cause of CKD is generally related to the presence of various kidney diseases, including lupus nephritis, antineutrophil cytoplasmic antibody‐associated vasculitis and renal transplantation, which are closely related to the activation of complement system.^12^ As reported, complement activation products (iC3b, Bb, and MAC) increased in the urine, and complement regulatory proteins (DAF and CD59) decreased in the kidney.^13^, ^14^ Moreover, MAC also deposited in kidney mesangium, vessels and tubules of patients and mouse models with a wide variety of kidney diseases.^15^, ^16^, ^17^, ^18^ Complement inhibition with monoclonal anti‐C5 in the treatment or prevention of antibody mediated rejection suggested that complement system was activated.^19^ Meanwhile, tubular epithelial cells produced virtually all complement proteins which were produced predominantly by the liver, and activated complement system on its apical surface via the alternative pathway.^20^ The concentrations of C3, complement factor B and C9 were also increased in the urine and cyst epithelium, suggesting activation of the alternative pathway.^21^, ^22^ Moreover, Pratt et al.^23^ demonstrated that wild‐type mice with intact serum complement activity did not reject allogenic C3‐deficient kidneys, underlying that kidney‐derived complement activation was a key mediator of renal injury. Further studies identified that the level of C4d, a biologically inert fragment cleaved from C4 through classical pathway, was increased after antibody binding to graft endothelium in kidney transplant.^24^ In addition, published study demonstrated that glomerular deposition of MBL was associated with the severity and prognosis in IgA nephropathy, suggesting the involvement of the lectin pathway.^25^ Taken together, complement system was activated in kidney diseases which contributed to VC.

### 2.2 The activation of complement system in atherosclerosis

Atherosclerosis is a consequence of a chronic inflammatory process induced by activation of macrophages, complement and T‐lymphocytes. Published studies revealed that complement components (C1q, C3c, C3d, C4, C9 and Bb)^26^ and complement inhibitory proteins [decay‐accelerating factor (DAF), factor H, CD59, CR1]^27^ deposited in the coronary artery wall of atherosclerosis patients. Meanwhile, complement components (C1r, C1s, C4, C7 and C8) were upregulated in atherosclerotic plaques, but the levels of regulatory inhibitors C1‐INH, DAF, C4BP, CD46 and CD59 in atherosclerotic plaques were not significantly increased when compared with normal arterial tissue.^28^ What is more, is that, the complement system was activated through classical, alternative and lectin pathways by oxidized low‐density lipoprotein (ox‐LDL), enzymatically modified low‐density lipoproteins (E‐LDL) and cholesterol crystals *in vitro* experiments.^29^, ^30^, ^31^ Further researches also demonstrated that ox‐LDL activated the classical complement cascade in ApoE‐deficient mice model.^32^ LDL receptor (LDLR)/C3‐deficient mice model was also utilized to identified that atherosclerotic lesions beyond the foam cell stage was strongly dependent on an intact complement system,^33^ and LDLR/DAF‐deficient mice model demonstrated the regulatory effect of DAF on C3 activation in the process of atherosclerosis.^34^ Furthermore, clinical cross‐sectional studies found that some complement factors were positively correlated with the severity of atherosclerosis and were used as novel circulating biomarkers of atherosclerosis, such as C5a and C3a.^3^, ^9^ Interestingly, Fehérvári et al.^35^ found the level of C3 was associated with the severity of atherosclerosis but not with arterial calcification in peripheral artery disease. Thus, it is worthy of further study to explore the role of complement system in VC.

### 2.3 The activation of complement system in DM

It is well established that DM and its organ damage are caused by prolonged hyperglycaemia, but the cellular and molecular mechanisms are still not fully understood. Experimental and clinical evidences support a strong link between the complement system and the pathogenesis of DM and DM complications. First, genetic studies showed association of allelic variants of complement proteins with autoimmune DM. Next, animal and human studies confirmed the activation of complement system by hyperglycaemia and glycation end products in DM.^25^, ^36^ A study analysed C4d, a marker of antibody‐mediated classical pathway, showed the activation of complement in type 1 DM (T1DM) progression.^37^ Studies also found increased level of MBL in T1DM mice model,^38^ and MBL double‐knockout mice model was utilized to demonstrate that complement system was activated through lectin pathway in T1DM.^25^ What is more, is that, a population‐based cohort revealed that plasma concentration of C3 was a risk marker for incidence of DM,^39^ and C3a, generated during proteolytic cleavage of C3 in complement activation, had insulin‐like effects and played role in insulin resistance.^40^ The increase of MAC and glycated CD59 suggested the activation of complement system, and contributed to the vascular complications in DM.^41^

### 2.4 The activation of complement system in aging

Aging is a process strongly integrated with chronic inflammation and metabolism. Recently studies have discovered the connection between complement activation and several aging‐related diseases, such as Alzheimer's and Parkinson's disease, multiple sclerosis and age‐related macular degeneration. A cohort of healthy volunteers (from birth up to 75 years) revealed that systemic protein C1q and C3 level increased with aging.^42^ There was also a positive correlation between C1q and aging‐induced arterial stiffness.^43^ Hence, these studies emerged the activation of classic pathway in physiological aging. N‐glycome, a biomarkers of biological aging and longevity, activated the lectin pathway of complement by binding to Fcγ receptors and formation of autoantibody aggregates.^44^ While, in two large cohorts of centenarians demonstrated that serum MBL concentration was significantly lower in centenarians as compared to the general population, and MBL protein bound to senescent IMR90 fibroblasts thereby causing cell lysis *in vitro*, suggesting that MBL may be protective.^45^ Shimazaki et al.^46^ also found complement factor B, an important component of alternative pathway, reduced the number of senescence‐associated‐β‐galactosidase positive cells *in vitro*. In addition, the levels of complement regulatory proteins CD59, CD46, DAF and CR1 were significantly lower in Alzheimer's diseases patients than controls.^47^ Moreover, previous studies found that C3 and C4 were positively correlated with age and inversely correlated with life expectancy in centenarians.^48^ Another study demonstrated that low levels of C3 delayed renal senescence,^49^ and that the use of Radix polygalae saponins to affect C3 expression could also extend the lifespan of *C. elegans*.^50^ It is clear that complement and aging are closely linked, but the role of complement components and aging‐related VC is unclear.

## 3 LOCAL SYNTHESIS OF COMPLEMENT IN VC

Although hepatocytes are still considered as the main source of complement secretion, recent studies have confirmed that almost every cell type can produce complement proteins. Notably, local complement synthesis has been found to be operative in many diseases, including atherosclerosis and kidney diseases. In atherosclerosis, complement receptors (CR1, CR2, CR3, gC1q‐R as well as C3aR and C5aR) were identified to be expressed in plaques, and the mRNA levels of C1r, C1s, C4, C7 or C8 were found to be upregulated, whereas local productions of regulators including CD46, C1–INH, C4BP, DAF and CD59 were also detected. Furthermore, intracellular complement activation about C5a during atherosclerosis was observed in monocytes and macrophages upon exposure to cholesterol crystals and LDLR‐deficient mice lacking myeloid‐specific C5aR1.^51^ Meanwhile, kidney endothelial cells and tubular epithelial cells produced virtually all complement proteins and activated complement system on its apical surface via the alternative pathway in kidney diseases.^20^, ^23^ More important, increased C3 production by clonally expanding Sca1^+^ smooth muscle cells during atherosclerosis has been shown to exert proatherogenic functions.^52^ Local production of C5a by vascular endothelial cells and expression of C5aR also accelerated the response of effector CD8^+^ T‐cell to heart transplant *in vitro* culture systems and *in vivo*.^53^ Thus, local complement production by resident cells of vascular system played a pivotal role in the development of VC. Collectively, local synthesis of complement is crucial but remains largely understudied in VC.

## 4 THE ROLE OF COMPLEMENT SYSTEM IN VC

**FIGURE 2:**
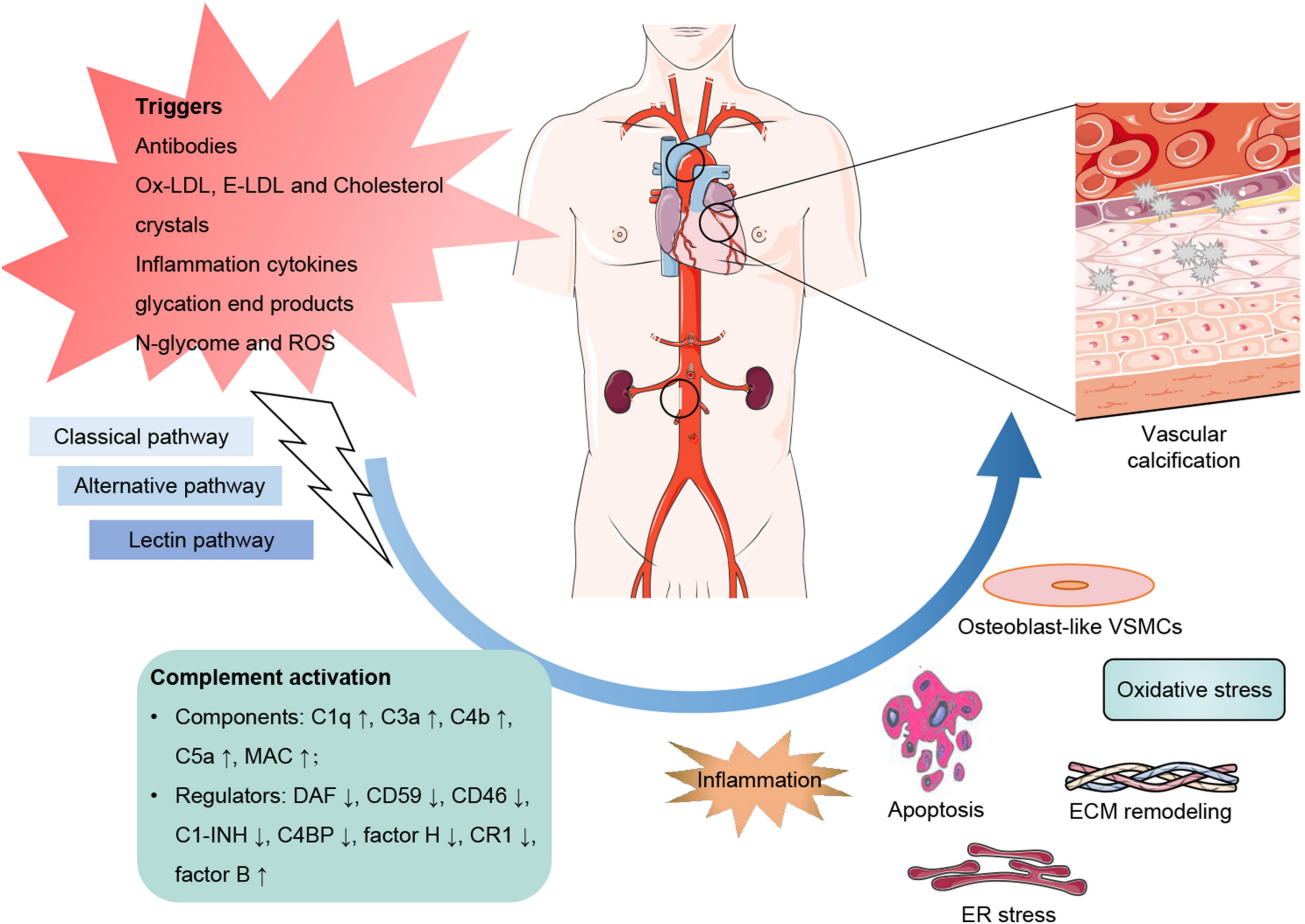
The complement system in VC. The complement system is activated by antibodies, modified LDL (Ox‐LDL and E‐LDL), cholesterol crystals, inflammation cytokines, glycation end products, *N*‐glycome and ROS through classical, lectin and alternative pathways in VC. The levels of complement components (C1q, C3a, C4b, C5a, and MAC) and regulator factor B increase, and the levels of regulatory factors (DAF, CD59, CD46, C1‐INH, C4BP, factor H and CR1) decrease in VC. Then, complement system may modulate the VSMCs osteogenic transdifferentiation, systemic inflammation, ER stress, ECM remodelling, oxidative stress, and cell apoptosis in the development of VC. ECM, extracellular matrix; E‐LDL, enzymatically degraded low‐density lipoprotein; ER, endoplasmic reticulum; MAC, membrane attack complex; Ox‐LDL, oxidized low‐density lipoprotein; ROS, reactive oxygen species.

As shown in a cross‐sectional clinical study, the level of C3a was positively correlated with abdominal aortic calcification in patients on haemodialysis,^7^ and this phenomenon was also observed in aortic and coronary calcification in middle‐aged women and patients with systemic lupus erythematosus.^54^ Meanwhile, the serum level of C5 was positive with coronary calcification in atherosclerosis patients.^9^ But, Fehérvári et al.^35^ explored that the level of C3a was not associated with arterial calcification in peripheral artery diseases according to general arterial calcification score and intima‐media thickness. Moreover, there was no association between C4 and aortic or coronary calcification in middle aged women.^8^ Taken together, complement components may take part in and play different roles in VC, but there are still no relevant research to explore its role and molecular mechanisms in VC. Accumulative studies have demonstrated that vascular smooth muscle cells (VSMCs) osteogenic transdifferentiation, systemic inflammation, endoplasmic reticulum (ER) stress, extracellular matrix (ECM) remodelling, oxidative stress, apoptosis and loss of mineralisation inhibitors, all contribute to VC.^55^ At the same time, the complement system was identified to regulate these processes *in vivo* and *in vitro*. Hence, we suspect that the complement system may play a role in VC through the following molecular mechanisms according to previous cumulative studies (Figure 2).

### 4.1 Complement system and VSMCS osteogenic transdifferentiation

Vascular smooth muscle cells osteogenic transdifferentiation is the crucial pathological basis for VC, which is manifested by the increase of osteogenic phenotype and the decrease of contractile phenotype. Although there was no research on the role of complement system in VSMCs osteogenic transdifferentiation, published researches have revealed the promotion of C5aR on the differentiation of human mesenchymal stem cell to osteoblasts, demonstrating by the increase of the osteogenic phenotype marker Runt‐related transcription factor 2. Furthermore, VSMCs osteogenic transdifferentiation is similar to physiological bone mineralisation which is regulated by complement system. As shown in osteoimmunological studies, C5a promoted nuclear factor kappa B ligand expression by binding to C5aR1 during osteoblast differentiation,^6^, ^56^ and mice with osteoblast‐specific C5aR1 overexpression displayed impaired fracture healing, while the number of osteoclasts in C5aR1‐knockout mice decreased and the bone mass significantly increased.^56^ Matsuoka et al.^57^ also demonstrated that C3a stimulated osteoblast differentiation *in vitro*. Besides, some fracture models were utilized to identified complement factors, including C3, CD59 and MAC, regulated physiological bone mineralisation.^58^, ^59^ Collectively, complement system may promote VSMCs osteogenic transdifferentiation in VC.

### 4.2 Complement system and systemic inflammation

Current evidences suggest that persistent low‐grade systemic inflammation is closely interrelated with and promotes VC, which is related with the increase of circulating inflammatory cytokines, such as C‐reactive protein (CRP), interleukin (IL)‐6, IL‐1β and tumour necrosis factor‐alpha (TNF‐α). Inflammatory cytokines induced endothelial‐to‐mesenchymal transition through downregulation of bone morphogenetic protein receptor type 2, inhibited the mobilisation and infiltration of bipotent mesodermal progenitor cells, and activated tissue‐nonspecific alkaline phosphatase by upregulating tissue‐nonspecific alkaline phosphatase activity to exacerbate VC.^60^, ^61^ In haemodialysis patients, a high CRP level was significantly related with VC in both the aorta and the radial arteries, and was a significant risk factor for abdominal aortic calcification progression.^62^ Meanwhile, C3a and C5a were positive correlated with CRP, and higher C3a level was independently associated with severe abdominal aortic calcification in haemodialysis patients.^7^ Although the role of complement system in infection has been well established, complement is becoming increasingly recognized as a key contributor to inflammation. As reported, C5a, C4a and C3a were demonstrated to increase the synthesis of IL‐1β, IL‐18 and TNF‐α through activating the NLRP3 inflammasome in neutrophils and monocytes,^63^, ^64^ while the complement regulatory protein CD46 decreased the expressions of pro‐IL‐1β and NLRP3 in epithelial cells.^65^ In addition, some studies found that persistent low‐grade systemic inflammation decreased the level of anti‐calcific molecule such as fetuin‐A, but the mechanisms need further exploration.

### 4.3 Complement system and ER stress

Increasing studies have shown that ER stress accelerates the development of VC by promoting osteogenic transdifferentiation, inflammation, autophagy, apoptosis and the release of matrix vesicles through its transducers, PKR‐like endoplasmic reticulum kinase (PERK), inositol‐requiring enzyme‐1 (IRE1α) and activating transcription factor 6 (ATF6) which are regulated by different ER stress activation molecules. What is more, is that, complement cascade activation occurs in the midst of a variety of cellular stresses that severely perturb ER stress.^66^, ^67^ In macrophages, C3 interacted with glucose‐regulated protein 94 and impacted M2 profile during ER stress.^68^ Meanwhile, C3a‐C3aR induced ER stress in mice exposed to cigarette smoke and RPE cells exposed to 5% cigarette smoke extract.^69^ C5a induced ER stress by activating the PERK and IRE1α pathways in neutrophils during the development of acute lung injury.^70^ What is more, is that, the MAC was identified as a key regulatory in ER stress, which induced damage to the membrane of the ER with the increased expression of glucose‐regulated protein 78 and glucose‐regulated protein 94 in glomerular epithelial cells.^66^ In addition, soluble DAF abrogated tunicamycin‐induced the expression of ATF6 and glucose‐regulated protein 78 during ER stress in alveolar type II epithelial cells.^71^ Taken together, abnormal increase or activation of complement system leads to ER stress, thus affecting VC.

### 4.4 Complement system and ECM remodelling

Recent advances demonstrate that ECM not only provides a scaffold for mineral deposition, but also acts as an active signalling entity in pathological VC.^72^ More importantly, complement system regulates ECM remodelling in various diseases. As shown in ApoE‐deficient mice model, C3 and C4 both bound to collagen in vascular wall which promoted vascular stiffness and atherosclerosis.^73^ MAC was also observed deposited in inflamed or sclerotic areas during the development of chronic nephritis, and stimulates collagen synthesis in human glomerular mesangial cells and glomerular epithelial cells.^74^, ^75^ Previous studies revealed that C5a and C3a promoted the expression of collagen and fibronectin which were the major components of ECM in renal fibrosis and subretinal fibrosis,^76^, ^77^ while DAF diminished the level of collagen *in vivo* and *in vitro*.^71^ Furthermore, in human macrophages, C5a induced the expression of matrix metalloproteinase (MMP)‐1 and MMP‐9 which were identified to promote the degradation of elastin.^78^ The above evidences indicate that complement system may act through regulate ECM remodelling in VC.

### 4.5 Complement system and oxidative stress

Oxidative stress emerges as a critical mediator to promote VC through several mechanisms, including phosphate imbalance, VSMCs osteogenic transdifferentiation, inflammation, DNA damage and ECM remodelling.^79^, ^80^ At the same time, studies have revealed the regulation of complement system on oxidative stress. In normal and asthmatic airway epithelium, complement regulatory factor CD46 inhibited oxidative stress.^65^ C3‐deficient mice were used to identify that C3 depletion ameliorated age‐dependent oxidative stress.^81^ Moreover, complement factor H inhibited oxidative stress in macrophages by binding malondialdehyde epitopes.^82^ Oxidative stress leads to oxidative damage, resulting from reactive oxygen species (ROS) generation that exceeds local antioxidant capacity. In polymorphonuclear leucocytes and hepatic ischemia–reperfusion injury mice model, the extracellular ROS production was stimulated by C5a–C5aR1,^83^, ^84^ and the production was also stimulated by C3a‐C3aR in human eosinophils.^85^ Notably, the complement system activation is also regulated by oxidative stress in different conditions, so it is interesting to explore the association between complement system and oxidative stress in VC.

### 4.6 Complement system and cell apoptosis

Vascular smooth muscle cells calcification model demonstrated the increase of cell apoptosis, while inhibition of apoptosis, for example by caspase inhibitors, significantly reduced calcification.^86^ Apoptotic bodies were thought to contain high concentrations of calcium and deposited on the ECM causing calcification. As for apoptosis, the regulatory effect of complement system is complex, due to the different cell types and disease backgrounds. As reported, C5a–C5aR promoted apoptosis in brain vascular endothelial cells of experimental lupus, lung cells of acute lung injury or murine kidney endothelial cells,^87^, ^88^, ^89^ but delayed apoptosis of human neutrophils via an extracellular signal‐regulated kinase and bad‐mediated signalling pathway.^90^ What is more, is that, C3a–C3aR induced apoptosis in acute pancreatitis rats model,^91^ but C3 prevented imiquimod‐induced bullous‐like skin inflammation by inhibiting apoptosis.^92^ In addition, MAC was found to co‐locate with apoptotic cells and cholesterol crystals in human arterial wall with atherosclerosis by immunoelectron microscopy and triggered apoptosis in glaucoma mouse model.^93^, ^94^, ^95^ As a result, the role of complement system in cell apoptosis is more complicated than simple magnification. Thus, there needs further study to explore the role of complement components in VC.

## 5 CONCLUSIONS

Vascular calcification contributes to the high morbidity and mortality of CVD in patients with CKD, atherosclerosis, DM and aging. Exploring the mechanisms of VC helps to provide a direction for slowing the progression of this burgeoning health concern. Our knowledge of the role of complement system in VC is relatively limited at this time. Nonetheless, there are growing number of evidences prove that complement system is involved in VC. It is crucial to understand the intimate interplay between the activation of complement system and the process of VC through *in vitro* and *in vivo* experiments. It would be interesting to investigate whether complement components contribute to VSMCs osteogenic transdifferentiation, ER stress or ECM remodelling in VC and complement regulator factors regulate these processes. VC mice models with specific deletion or overexpression of complement genes will be also utilized to explore the role of complement components in VC, hence, providing an appealing therapeutic target for VC. Furthermore, therapeutic agents, targeting on complement activation cascade, are promising for VC which have been used for some diseases. As reported, high‐dose IVIg targeted on C3 is used in dermatomyositis patients,^96^ eculizumab (a C5 blocker) is used in paroxysmal nocturnal hemoglobinuria, atypical haemolytic uremic syndrome, neuromyelitis optica and myasthenia gravis^97^ and avacopan (a specific C5aR1 antagonist) is used in antineutrophil cytoplasmic antibody‐associated vasculitis.^98^

## AUTHOR CONTRIBUTIONS

**Aiting Liu:** Conceptualization (equal); writing – original draft (lead); writing – review and editing (lead). **Pei Luo:** Writing – review and editing (supporting). **Hui Huang:** Conceptualization (equal); funding acquisition (lead); writing – original draft (supporting); writing – review and editing (supporting).

## FUNDING INFORMATION

This work was supported by National Key Research and Development Program (2020YFC2004405), National Natural Science Foundation of China (82270771, 82061160372 and 81870506), Central Military Commission Key Project of Basic Research for Application (BWJ21J003), Regional Joint Funding Key Project of Guangdong Basic Research and Basic Research for Application (2021B1515120083), Key Project of Sustainable Development Science and Technology of Shenzhen Science and Technology Innovation Committee (KCXFZ20211020163801002), Basic Research Project of Shenzhen Science and Technology Innovation Committee (JCYJ20190808102005602), Futian District Public Health Scientific Research Project of Shenzhen (FTWS2022001), Chinese Association of Integrative Medicine‐Shanghai Hutchison Pharmaceuticals Fund (HMPE202202), and Shenzhen Key Medical Discipline Construction Fund (SZXK002) to HH.

## CONFLICT OF INTEREST STATEMENT

The authors confirm that there are no conflicts of interest.

## Data Availability

Data sharing not applicable–no new data generated.
